# Developing a Culturally Adapted Digital Health Application for Older Hispanic Adults With Type 2 Diabetes: Protocol for a Qualitative and Pilot Study

**DOI:** 10.2196/76294

**Published:** 2026-01-23

**Authors:** Joshua Caballero, Raymond L Ownby, Henry Nolan Young, Michelle B McElhannon, N M Mahmudul Alam Bhuiya, Kenny H Esho, Russ H Palmer

**Affiliations:** 1 Department of Clinical and Administrative Pharmacy, College of Pharmacy, University of Georgia, 250 West Green Street, Athens, GA, 30602, United States, 17065421132; 2 Department of Psychiatry and Behavioral Medicine, Dr. Kiran C. Patel College of Osteopathic Medicine, Nova Southeastern University, Fort Lauderdale, FL, United States; 3 Division of Experience Programs, College of Pharmacy, University of Georgia, Athens, GA, United States; 4 Athens Neighborhood Health Center, Athens, GA, United States; 5 Division of Instructional Innovation and Research, College of Pharmacy, University of Georgia, Athens, GA, United States

**Keywords:** pharmacist, diabetes, digital app, health literacy, Hispanics, Latinx

## Abstract

**Background:**

Over 40% of Hispanic individuals have below basic health literacy levels, significantly impacting their ability to manage chronic conditions such as type 2 diabetes (T2D). Chronic disease self-management, essential for improving adherence, is often hindered by modifiable factors such as health literacy. Ambulatory care clinical (ACC) pharmacists, through comprehensive medication management (CMM), play a critical role in addressing these barriers; however, current methods for delivering CMM vary significantly, limiting effectiveness. Digital technology may offer significant potential for improving medication adherence, particularly among Hispanic individuals who use smartphones to seek health information.

**Objective:**

This study aims to revise, develop, and pilot test a culturally adapted, individually tailored digital health app designed to enhance health literacy and medication adherence among older Hispanic adults with T2D by integrating interactive educational modules into pharmacist-delivered CMM.

**Methods:**

Our team previously developed a prototype computer-delivered, culturally adapted intervention targeting health literacy and adherence among older Hispanic individuals. The proposed project involves transforming this intervention into a modernized digital health app. We will achieve 2 specific aims. The first aim is to refine and modernize previously developed content into an interactive digital health app suitable for older Hispanic adults with T2D and ACC pharmacists. A total of 20 patients and 10 ACC pharmacists will review and provide feedback on the app modules. Usability and acceptability will be measured using validated tools, including the System Usability Scale, Adjectival Ease of Use Scale, and the technology acceptance model. The second aim is to conduct a pilot test with 40 Hispanic adults (aged ≥50 years) diagnosed with T2D. Participants will be recruited from a local Federally Qualified Health Center where ACC pharmacists manage diabetes care. Patients will complete a baseline CMM session, interact with the app modules, and return for 1 or 2 CMM follow-up visits. We will assess medication adherence, hemoglobin A_1c_, and fasting glucose levels as primary outcomes.

**Results:**

The digital app has been modernized and completed for acceptability and usability testing. We will begin testing the hypothesis that the app will have above-average usability and acceptability (System Usability Scale: mean score ≥71 and Adjectival Ease of Use Scale: mean score ≥5), with an overall goal that the digital app will be acceptable (via the technology acceptance model) by ≥90% of the patient participants and ACC pharmacists. In addition, anticipated health outcomes for aim 2 include improvements in medication adherence (>80%), hemoglobin A_1c_ (≥0.75% reduction), and fasting glucose (≥25% reduction) from baseline.

**Conclusions:**

When the study is completed, it is expected that the culturally adapted digital health app will be acceptable and usable among both patients and pharmacists. Successful results will enable wider dissemination of the refined app and establish a framework adaptable to other chronic diseases, diverse languages, cultures, and health care settings.

## Introduction

### Epidemiology

The Centers for Disease Control and Prevention estimates that over 34 million people in the United States have diabetes [1]. Readmission data from the Centers for Medicare and Medicaid Services show that patients with diabetes as a comorbidity need to be targeted for high-cost and disease management [2]. Diabetes has the third-highest 30-day all-cause readmission rate among Medicaid patients, resulting in nearly 34,000 readmissions and at a cost of approximately US $471 million [3]. In addition, the prevalence of diabetes is higher among minority groups (eg, Hispanic populations). Data show that state-insured Hispanic individuals with type 2 diabetes (T2D) have poorer glycemic control and adherence to newly prescribed diabetes medications (oral or insulin) compared to non-Hispanic White populations [4]. Diabetes medication adherence rates also continue to decrease in the United States. Data suggest that patients with diabetes who had an unscheduled readmission within 90 days were more likely to be lower-income minorities on public insurance [5,6]. As a result, minority patients with T2D are more likely to have poorer health outcomes, higher risk of hospitalizations, and higher economic burden [1,2,7].

### Tailored Information and Multimedia Instruction as Interventions

The primary method of provider-patient communication about medications has traditionally been brief oral communications during often hurried medical consultations. Studies suggest that patient retention of information in this context is poor, often less than 50% [8]. The introduction of multimedia presentation of material may increase learning and retention and may be helpful for various conditions and populations [9-12]. Unfortunately, the results of using eHealth interventions for T2D have been mixed, including those focused on Hispanic populations [13-15]. Limitations that commonly plague eHealth interventions in T2D are (1) lack of cultural adaptation for minorities (such as Hispanic individuals); (2) unknown usability or operability by patients and providers; (3) educational health literacy component that is predominantly mono-directional; (4) lack of tailoring to the individual; (5) education principles of multimedia learning are not used; (6) lack of general medication information, or such information is provided by nonspecialists; (7) cost-prohibitive; and (8) lack of sufficient security of patients’ personal health information [13-15]. A recent National Institutes of Health (NIH)-funded project (R01DK112322) developed an eHealth intervention using text messaging that achieved a notable 1% reduction in hemoglobin A_1c_ (HbA_1c_) but did not show improvements in medication adherence [14,16]. While promising, the intervention encountered certain practical challenges, such as limited capacity for individual tailoring and potential security concerns associated with a cloud-based platform [16]. Accordingly, our digital health app aims to address these factors and build on the progress shown by previous interventions.

### Hispanic Smartphone and Digital Health Information Use

Similar to non-Hispanic White people, approximately 85% of Hispanic people use a smartphone daily [17]. Hispanic adults are outpacing all other groups in smartphone use growth rate. In addition, similar to other groups, Hispanic adults spend approximately 3 hours per day on a smartphone and 1 hour per day on a tablet. Hispanic individuals are more likely to use smartphones for health-related information compared to non-Hispanic White or Black populations (73% vs 58%‐67%) and more likely to view educational content (45% vs 26%‐32%) [18]. Unfortunately, misinformation across many health topics (eg, noncommunicable diseases and medical treatment) ranges between 30% and 87% [19]. In addition, the ability to interpret information could be a problem, as identified by Powell et al [20], who found that patients often misinterpret online reputable diabetes information (ie, American Diabetes Association myths). The proposed study will enable the investigators to use available technology to provide patients access to correct information, while also enabling health care providers to assess patient interpretation of the information to ensure patients understand. The use of digital apps has become common among all populations in the United States, with Hispanic individuals reporting using an average of approximately 27.9 apps and spent 41.5 hours using mobile applications per month. [21]. Therefore, Hispanic individuals may be more likely to use a digital health app to educate themselves. The digital health app proposed may improve T2D health literacy, promote patient engagement, and allow for precision counseling. Precision counseling is a personalized approach to patient education and adherence that considers social and environmental stressors, medication side effects, and health literacy to tailor interventions for each patient [22]. Similar to precision medicine, it leverages technology to optimize counseling strategies, provide more effective treatment, and potentially improve health outcomes.

### Pharmacists’ Comprehensive Medication Management (CMM) Services

Data show that health care providers may not adequately promote medication adherence due to a lack of time or by failing to use an effective communication format for the patient (eg, appropriate reading level, language) [23]. There is an increasing role for pharmacists in assisting patients with T2D. Section 10,328 of the Affordable Care Act amended section 1860D-4(c)(2)(ii) of the Act and requires prescription drug plan sponsors (Part D plans) to offer an annual medication review as part of the comprehensive medication management (CMM). CMM is a standard of care that guarantees each medication is individually assessed for appropriateness, effectiveness, and safety for the individual patient. Using CMM can improve chronic disease self-management and related outcomes. Some data suggest pharmacists can positively impact adherence and decrease HbA_1c_ (a measure of blood glucose control over time) through CMM [24]. However, other studies on the benefits of CMM are inconclusive because of limitations or failure to demonstrate efficacy in chronic disease self-management [25,26]. In these studies, researchers have used digital interventions that may not be readily comparable or optimally defined [24,25]. Limitations of these studies have included time constraints, lack of Hispanic populations, lack of integration into normal workflow, and absence of innovative digital eHealth technology (eg, individual tailoring) [27,28]. Another key limitation in these studies is their focus on adherence as an isolated problem. Given that factors affecting adherence and health outcomes are multidimensional, the content we developed and culturally adapted is multifaceted [22]. It is based not only on providing tailored content, but also on assessing modifiable factors of adherence (eg, depression). As a result, it can enable pharmacists to provide precision counseling to influence areas in which a patient shows a deficiency and be modified based on various factors of adherence.

### Synopsis of Current Culturally Adapted Intervention

We have demonstrated that health literacy is impacted by cognitive abilities, academic skills, and health knowledge [29]. Based on these results, we developed and culturally adapted a health intervention for older Hispanic adults with T2D using Adobe Captivate [22]. This intervention suggests favorable results regarding usability among patients and pharmacists providing CMM. Overall, there are 3 modules that provide information, verify understanding of content with queries, and allow the patient to tailor their education (eg, knowledge about medications they take). There are also queries on other factors impacting adherence (eg, depression and cost) that will assist the provider in addressing or referring the patient to other services (eg, mental health support). This differs from other digital technologies that do not incorporate all these measures. For example, some tools take an innovative approach by using issue cards to explain medication classes but do not appear to verify knowledge gained or explain how the tool can be linked to prescribers managing their diabetes in a simple format [30]. Another novel digital app focused on Spanish speakers sends 2‐3 motivational texts per day, but also does not appear to collect knowledge gained or provide specific information regarding their medications [14,31].

### Aims and Hypotheses

There are 2 aims in the proposed study. Aim 1 consists of refining and modernizing developed diabetes content from our previous study [22], developing the Patient and Provider apps, and testing the usability and acceptability by patients and pharmacists. The Aim 1 hypothesis is that the digital apps (ie, Patient and Provider apps) will have above-average usability and acceptability (System Usability Scale [SUS] mean score ≥71 and Adjectival Ease of Use Scale [AEOUS] mean score ≥5) with an overall goal that the digital apps will be acceptable (Davis’ technology acceptance model [TAM] ≥90%) to the participant patients and pharmacists.

The goal for Aim 2 is to pilot test the digital health apps to assess adherence and health outcomes in 40 Hispanic patients with T2D. The Aim 2 hypothesis is that the digital health apps will improve adherence (>80% average adherence for the individual), HbA_1c_ (≥0.75% reduction), and fasting glucose (≥25% reduction) from baseline.

## Methods

### Study Design Overview

**Figure 1.**
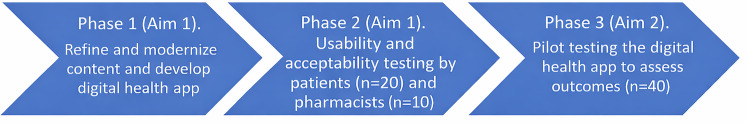
Development of patient and provider diabetes digital health apps.

The study consists of 3 phases, with Phase I and II addressing Aim 1 and Phase III addressing Aim 2 (Figure 1). During Phase I, the previously developed content will be refined and modernized. Both digital health apps will also be developed. In Phase II, the usability and acceptability testing will be completed for patients and pharmacists. During Phase III, Aim 2 will be assessed by pilot testing the digital health app to assess medication adherence and health outcomes.

### Phase I

During this phase, we want to inform the design of the digital health app with evidence-based instructional principles. Therefore, to maximize the effectiveness of the digital health app, the content should promote and embrace effective learning. As a result, content will be refined to ensure the educational component is bidirectional, educational principles are used to maximize multimedia learning, general medication information is included, and cost and security concerns are addressed. Overall, the content will be improved for digital learning. The content will then be transferred into the Patient digital health app, containing 3 modules. While developing the content, we will adapt Gagne’s Nine Events of Instruction (GNEOI) as an overarching evidence-based strategy to support the effectiveness of the new app [32]. GNEOI is aligned with best practices derived from cognitive psychology and information processing theory. It includes the following sequence of events for organizing learning: (1) gain learners’ attention, (2) inform learners of objectives, (3) stimulate recall of previous knowledge, (4) present the content, (5) provide learning guidance, (6) elicit performance, (7) provide feedback, (8) assess performance, and (9) enhance retention and transfer. Within the overarching GNEOI strategy, our team will modernize and improve the multimedia learning of the app by tailoring the content following Mayer’s 12 principles of cognitive theory of multimedia learning [33]. These principles, also firmly grounded in information processing theory, include providing text, graphics, and narration that support learning goals, keeping visuals simple, avoiding distractions (eg, unnecessary background music), using human voiceover, and adhering to the redundancy principle (eg, allowing the patient to select graphics and narration vs graphics and text). We will use whiteboard animation software to convert written content into approximately 30‐60–second videos. Whiteboard animation consists of using simple animation to teach concepts that follow many of Mayer’s 12 principles. Data suggest whiteboard animation can assist with increasing patient and caregiver information retention [34]. These animations can be tailored to different populations (eg, by changing skin tones or language and adding closed captioning). By following Mayer’s 12 principles, the content will support knowledge construction in which the patient is building a coherent mental representation from the information presented. Please note that for Phase I, no participant recruitment will be needed.

#### Module Content

**Figure 2.**
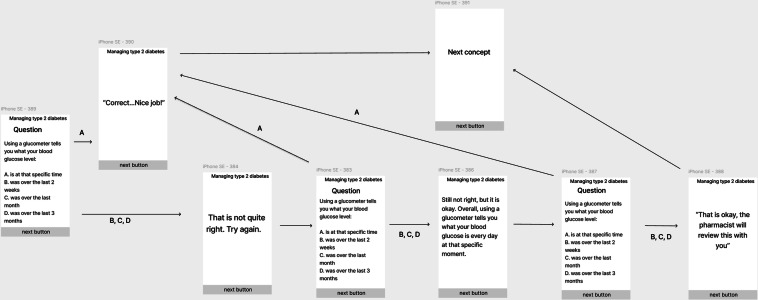
Bidirectional learning model.

There are 3 modules guided by the enhanced information-motivation-behavioral (IMB) model for diabetes, which allows for bidirectional learning and fits squarely within the overarching GNEOI strategy. The IMB skills model provides an actionable plan to design and implement digital health interventions [35,36]. For example, the patient is asked a question about information they just saw or read. If they answer correctly, the module congratulates them and moves on to another topic; if they do not, the module summarizes the content in a follow-up slide or video and asks the question again. (Figure 2). Overall, the IMB model proposes that if individuals receive the information they need, feel motivated to modify their actions, and apply the necessary skills, it may provide sustained behavior change that promotes adherence and health outcomes [35,36].

The first module provides general information on T2D (eg, the role of insulin) and asks questions about mental health and family support. The second module provides information on diet, exercise, the benefits of medication adherence, and methods of monitoring T2D. The module also asks the patient if there are specific areas they would like to explore further (eg, diet, exercise, vaccinations, and glucometer use). For example, the module will not only discuss the benefits of using a glucometer but will also ask if they use or need one, thereby alerting the pharmacist of the need to secure one for the patient before the next counseling session. The third module allows patients to select their prescribed medications, provides information about those medications, and asks about their ability to administer them. For example, if a patient states they take insulin, the module will provide information about insulin and then ask if they have fears about injecting it and if they would like additional instructions on proper administration. This information will allow the pharmacist to develop an individualized CMM plan focused on specific needs for each patient.

After the content is refined and modernized, 2 diabetes experts and an instructional design expert will review it and provide feedback. The content will then be updated based on their input, and the app developer will create 2 digital apps: the Patient and Provider apps. The Patient app will house the 3 modules. Patients will be able to easily download it to a mobile device using the app stores on iOS (eg, Apple iPhone) or Android (eg, Google phone). Based on preliminary discussions with the app developer, the app will fit into a 16×9 (1920×1080 pixels) aspect ratio and can be adapted to various formats (eg, smartphone and tablet). A narrated voiceover option will also be included. Therefore, the app will be accessible to patients who may be illiterate or have some visual problems (eg, mild-moderate diabetic retinopathy).

After the patient downloads the digital app and completes the 3 modules, the app will encrypt the patient’s responses and generate a QR code. QR codes are used to package and transmit information securely, and their use has become more widespread, even becoming a recent function on Windows, allowing a user to convert a webpage into a QR code. QR codes provide an effective and secure method of transmitting patient responses directly to a provider who uses the Provider app. The QR code will contain all the encrypted information captured from the patient’s responses and does not have to be uploaded to the cloud, where the information could be compromised. More importantly, the patient’s responses contained within the QR code cannot be decrypted without the Provider app. The patient will have the option to send their QR code via email ahead of time or provide it at their follow-up appointment. The Provider app will be used by the clinic to decrypt the data stored in the QR code using a concept in computer science known as asymmetric cryptography, which is the industry standard. This technology allows for the creation of a lock made of an unpredictable, large, and randomized series of numbers embedded in the QR Code, which can only be decrypted using a private key (eg, the Provider app). Once the QR code is scanned by the clinic pharmacist using the Provider app, the pharmacist will be able to access the patient’s responses, presented in a user-friendly format. Although the modules and patient responses in the Patient app will be in Spanish, the information will be available in the Provider app in either English or Spanish according to the pharmacist’s preference.

### Phase II

During this phase, usability and acceptability testing by patients and pharmacists will be completed. To screen the patients, we will assess their language dominance using the Language Experience and Proficiency Questionnaire [37]. We will assess the patients’ acculturation level using the scale developed by Marin et al [38]. This 12-item acculturation scale has been widely used in research on Hispanic individuals. Acculturation, as measured by this scale, correlates highly with respondents’ generation, length of residence in the United States, age at arrival, and ethnic self-identification. We will use criterion sampling to recruit up to 20 older Hispanic patients with T2D and low acculturation.

#### Patient Usability and Acceptability Testing

A recent systematic review of usability research in educational technology found that many studies are narrowly focused and insufficiently iterative, leading to results that fail to support the user experience as comprehensively as possible [39]. A frequent problem is the application of unimodal approaches that apply a single questionnaire to obtain a summative measure of overall quality. In contrast, our patient usability and acceptability testing will apply a robust set of methods intended to iteratively improve the quality of the design, promoting a highly usable and acceptable experience. This will include repeatedly triangulating results from cognitive “think-aloud” interviews, screen recordings, and the previously validated SUS and AEOUS [40,41]. A previously used questionnaire based on Davis’ TAM will also be used to evaluate system acceptability [22,42]. In summary, a set of 5 patients (seen separately) will be observed interacting with the Patient app and will be asked to “think out loud” so that their cognitive processes can be tracked and areas of difficulty can be identified as they review the modules [43,44]. Simultaneously, their screens will be recorded, and transcripts will be produced and timestamped for analysis. After each session, the SUS, AEOUS, and TAM will be administered to provide a snapshot of system quality. The app and content will be revised based on these results, tested with a new group of 5 participants (seen separately), and revised further [45]. This process may be repeated until no further difficulties are identified and benchmark SUS, AEOUS, and TAM scores are obtained, potentially eliciting responses from 15 to 20 participants [46,47]. Previous literature has indicated that small convenience samples are highly effective in software studies [48]. A sample size of 10 should be sufficient based on previous experience [22]; however, since we will also be evaluating the app and content on different devices, we will target up to 20 participants.

#### Pharmacist Usability and Acceptability Testing

We will test the usability and acceptability of the intervention for incorporation into CMMs with up to 10 pharmacists. Pharmacists will be recruited from our collaborative clinic (Athens Neighborhood Health Center) and other federally qualified health centers that work with Hispanic patients and the CMM process. We will conduct individual interviews with the pharmacists to test the intervention and obtain feedback (eg, SUS, AEOUS, and TAM scores) to improve the ease and practicality of use when conducting CMM. They will also test and evaluate the Provider digital app to identify the optimal method of displaying patient responses. Our previous study tested much of the content with pharmacists and required 1 round (5 individual interviews) [22]. However, since we are also focused on developing the digital app for pharmacists, we budgeted for 10 pharmacists in case 2 rounds of testing are needed to make sure the Provider app is user-friendly.

### Phase III

The third phase will test health outcomes: medication adherence, HbA_1c_, and fasting blood glucose. During this phase, the pharmacist will use the digital health app with 40 adult Hispanic patients with T2D who meet the inclusion requirements (stated under the ”Recruitment” section). The study will take place at the Athens Neighborhood Health Center. If by 6 months, we have not recruited at least 25 patients, we will pursue another local federally qualified health center. After consent, patients will be instructed on downloading the digital health app, completing the modules within the first 2 weeks, and generating the QR code. Their preference for either submitting the QR code via email or bringing it to their next appointment will be confirmed. Medication adherence will be measured using scales previously used by the investigators (the Gonzalez-Lu questionnaire and the visual analog scale) [49,50]. The Gonzalez-Lu questionnaire and visual analog scale are being used due to their ease of use and strong reliability and correlation to electronic measurements of medication adherence [51-53]. Baseline HbA_1c_ and blood glucose levels (fasting) will be measured via labs conducted as standard medical practice. A follow-up visit will occur 3‐4 months later. Depending on patients’ responses, an additional visit 3‐4 months later may be needed. The app would immediately assist a patient who is nonadherent due to not knowing how to take a medication (eg, the app would explain that taking metformin with food optimizes effectiveness and lowers side effects). However, a patient may be nonadherent due to the inability to pay for a particular medication; this would be captured by the app but not addressed immediately because they chose to provide their QR code at the follow-up appointment and not when the issue arose. In such a scenario, the patient would have a follow-up visit an additional 3‐4 months after appropriate changes are made (eg, selecting another medication). In summary, Phase III will test our Aim 2 hypothesis that the app will improve medication adherence (>80% average adherence for the individual), HbA_1c_ (≥0.75% reduction), and fasting glucose (≥25% reduction) from baseline. These reductions are reasonable target endpoints based on the literature [54-56].

### Recruitment

Two types of participants will be recruited for Phase II: patients and pharmacists. Patients (n=20) asked to participate will be Hispanic men and women aged 50 years and older with T2D who have been on diabetes medications for at least 90 days but who are not adherent (ie, <80% adherent) and have increased diabetes markers (ie, HbA_1c_ >7% and fasting glucose >125 mg/dL). In addition, they should be able to meaningfully participate in the informed consent process, demonstrate poor acculturation based on the Marin Acculturation Scale, be comfortable speaking Spanish (during the preconsent or consent process), have and use a smartphone or tablet, and be willing to participate in a single visit lasting approximately 90 minutes. Exclusion criteria will include cognitive or psychiatric difficulties so severe as to make the person unable to meaningfully participate in the informed consent process, diagnosis of Alzheimer disease or any other dementia, or receiving pharmacotherapy for Alzheimer disease, and severe retinopathy or visual impairment that impacts the ability to see module content on a smartphone or tablet. Pharmacist participants (n=10) asked to participate will be licensed pharmacists providing CMM to Hispanic patients with T2D in an ambulatory care setting. In addition, they should be able to meaningfully participate in the informed consent process and be willing to participate in a single visit lasting approximately 90 minutes.

Only patients will be recruited for Phase III. Patients asked to participate will be Hispanic men and women aged 50 years and older with T2D who have been on diabetes medications for at least 90 days but who are not adherent (ie, <80% adherent) and have increased diabetes markers (ie, HbA_1c_ >7% and fasting glucose >125 mg/dL). Inclusion criteria will be similar to those in Phase II. However, they must be willing to participate in a baseline office visit and 1‐2 subsequent follow-up office visits lasting 60‐90 minutes, with each visit spaced 3‐4 months apart. Exclusion criteria are similar to those described in Phase II.

### Measures

Data to be collected include participants’ demographics (eg, age, gender, and country of origin), self-reported medication adherence (eg, Gonzalez-Lu questionnaire and visual analog scale), and laboratory values (eg, HbA_1c_ and fasting glucose) from the clinic chart. Most self-reported data will be collected on paper and transferred into electronic databases secured on university-issued password-protected computers. Data will only be linked to participants’ identities by way of an identification number, and no personally identifiable information other than the identification number will be included with the data. The correspondence between participant identification numbers and their identity will be kept separately. All other data will be kept on password-protected university computers. In addition, during Phase II, individual software testing sessions will be recorded, with computer screen recordings of participant behavior (patients and pharmacists), while notes are taken by the investigator. These recordings will also be kept on password-protected university computers and will only be associated with the identification number and not with any personally identifiable information. Any recordings on software (eg, Lookback Usability Testing software) are also password-protected and will not include any protected health information. Lookback Usability Testing software provides the ability to assess usability by recording the participants’ interaction with the digital intervention. During Phase III, the pharmacists who are providing care to patient participants will have the ability to decrypt the QR code and match the patient with the identifier. This will allow the pharmacist to place the electronic file into the patient’s protected folder in their electronic health records. These records are password-protected and housed on password-protected computers. Please note this is not any different than the standard of care and normal operating procedures when medical records are received from other sources and securely uploaded to the patient’s electronic record.

### Procedures

Before the recruitment process, University of Georgia Institutional Review Board (IRB) approval for the proposed human participants research protocols will be secured, as well as any review required by the Athens Neighborhood Health Center. The study will also be registered on the United States government clinical trials website.

Overall, potentially eligible patient participants (for Phase II and III) will be recruited from patients at the Athens Neighborhood Health Center during their regular appointments. In addition, bilingual flyers will be posted in the clinic’s waiting room. Patients will be able to contact the investigator or clinician via email or phone call in English or Spanish to obtain more information about the study and/or express their interest in the study. Once patient participants are identified, a telephone screening or face-to-face interview in Spanish will be conducted for each potential participant. During the screening interview, participants will be informed of the nature of the study and may participate if interested and if the eligibility criteria are met. If they choose not to participate, they will be thanked for their interest and reminded that not participating does not impact their standing with the clinic. Potential pharmacist participants for Phase II will be recruited from the clinic site and locally (in the state of Georgia), as well as from other states serving Hispanic populations. As a result, it is important to note that pharmacist participants may be remotely interviewed via a secure server (ie, Zoom Meetings software) during Phase II. While pharmacists will have the option for remote interviews, visits for patient participants will all be conducted in person (face-to-face).

#### Aim 1

The primary endpoint of Aim 1 is to have a digital app that is user-friendly to both the patient and pharmacist. Regardless of whether the interview is face-to-face (for patients) or virtual (for some pharmacists), the software used (Lookback and Atlas.ti [Lumivero]), along with the recorded interviews, will allow us to capture information needed to conduct a thorough analysis. For each group of 5 participants, qualitative data (interviews and screen recordings) will be coded to identify features of the intervention that benefited or hindered the system’s ease of use and participants’ user experiences. Analysis will be based on an existing coding framework designed for medical interventions and expanded based on usability standards [38,55,56]. Analyst triangulation will be conducted to ensure high intercoder agreement. Issues found to have negatively impacted user experiences will be resolved before testing with new groups of 5 participants (for both patients and pharmacists). In addition, the SUS, AEOUS, TAM, and time to complete will be analyzed using descriptive statistics to provide quantitative snapshots of system quality for each group. This process of qualitative and quantitative analysis may be repeated with groups of 5 participants until no further difficulties are identified and benchmark scores are achieved for the SUS, AEOUS, and TAM. In summary, phase II will iteratively refine the digital apps based on qualitative analyses and test our Aim 1 hypothesis that the apps will have above-average usability and acceptability (SUS: mean score: ≥71 and AEOUS: mean score ≥5) with an overall goal that the digital apps will be acceptable (via TAM) by ≥90% of the patient participants and ambulatory care clinical pharmacists. These measurable targets are based on published data [40,57,58].

#### Aim 2

The primary endpoint of Aim 2 is to improve adherence and health outcomes. We completed power analyses for this aim using a mixed-effects simulation routine in Power Analysis Sample Size (PASS 16; NCSS, LLC) software, and drawing effect size estimates from the literature on the impact of pharmacist counseling on diabetes control [59,60]. This analysis shows our planned sample size of 40 will provide a power of 0.85 to detect a significant change from baseline in outcome measures. Outcome measures include improvements in adherence and a reduction in HbA_1c_ and fasting glucose. We will evaluate differences before and after the intervention (eg, 3‐8 months later) using repeated-measures mixed-effects models, enabling us to evaluate not only the significance of change but also the impact of key covariates (age, gender, and health literacy) and effect size for use in planning a larger trial.

### Evaluation Outcomes

After completion of Phase III, we will have produced 2 refined digital health apps. The Patient app will house the diabetes content for the patient to review and complete, while the Provider app will be able to transform the patients’ responses to the modules into an easy-to-read, pragmatic format to provide precision counseling. As a result, both apps will be ready for deployment and testing in a larger-scale study using a control group. The study will also provide a foundational framework for application to different diseases, languages, and cultures, and health care providers, potentially having a wider impact. This study will provide the foundation for our team to conduct a large-scale, robust, and pragmatic trial of the integration of the Patient and Provider apps into ambulatory care clinical pharmacist patient care services in geographically diverse federally qualified health centers to improve T2D medication use and outcomes. We plan to pursue such a larger-scale study, estimated to last 5 years via the R01 funding mechanism, to test the longer-term impact of the apps on health outcomes. In future studies, based on patient responses to these modules, follow-up visits, and as determined by the health care providers, we plan to implement artificial intelligence technology to deliver tailored prompts (via texts) to maintain and promote patient engagement and activation.

### Ethical Considerations

All recruitment materials will be provided to the University of Georgia IRB Committee for approval. Study personnel will complete all necessary training in the protection of human participants in research as required by the IRB and available through the Collaborative Institutional Training Initiative program. Patients and pharmacist participants will provide written informed consent obtained by trained study personnel before being enrolled in the study. All protected health information will be maintained in accordance with institutional regulations and policies, and standard ethical practice. The Phase I portion of the study did not require IRB approval since it involved modernizing and refining content. Phase II (patient and pharmacist participants’ qualitative analysis) and Phase III (pilot study assessing patient health outcomes) will require University of Georgia IRB approval. Informed written consent will be secured from all participants and appropriately stored.

## Results

The tailoring of content and video production has been completed. However, we received notice that our NIH funding was terminated in early May 2025 with the following explanation: “It is the policy of NIH not to prioritize research programs related to DEI (diversity, equity, and inclusion): research programs based primarily on artificial and nonscientific categories, including amorphous equity objectives, are antithetical to the scientific inquiry, do nothing to expand our knowledge of living systems, provide low returns on investment, and ultimately do not enhance health, lengthen life, or reduce illness. Worse, so-called DEI studies are often used to support unlawful discrimination based on race and other protected characteristics, which harms the health of Americans. Therefore, it is the policy of NIH not to prioritize such research programs.*”* As a result, we appealed the decision, stating that we will open the study to all races and ethnicities through a no-cost extension. The appeal was denied in late August 2025. In light of the NIH funding termination, the researchers have used their faculty development funds to continue their work on a smaller scale and have continued to seek other support mechanisms. As of January 2026, submission to the IRB has been completed to begin the qualitative (acceptability and usability) portion of the study (Phase II and Aim 1) in Hispanic adults with T2D and pharmacists. We expect acceptability and usability studies to be completed within 12 months. We anticipate that both digital apps will be acceptable and usable for both patients and pharmacists. In addition, during Phase III (Aim 2), we expect the digital apps to assist patients in improving their health outcomes. Specifically, we expect that the apps will improve adherence (>80% average adherence for the individual), HbA_1c_ (≥0.75% reduction), and fasting glucose (≥25% reduction) from baseline. These reductions are reasonable target endpoints based on the literature [54-56].

## Discussion

### Principal Findings

The research project aims to refine and modernize diabetes content into an interactive experience for patients, which will be aimed at developing tailored information. The responses provided by the patient will be provided to the pharmacist in an easy-to-read format that will allow the clinical pharmacist to gather the needed information to provide precision counseling to their patients.

Based on a previous study we completed, 5 themes among older Hispanic participants with T2D were identified, which included: financial considerations, polypharmacy, social and family support, access to medication information, and loneliness or sadness [22]. These themes are being applied to the current digital apps. For example, content and items on social and family support and loneliness or sadness are being implemented. In addition, the Patient app will provide brief information about loneliness and sadness and will have subsequent items exploring patients’ mental health (ie, Patient Health Questionnaire–2 [PHQ-2]). If a patient scores high on the PHQ-2 items, after decryption, the Provider app will alert the pharmacist to explore this area with the patient and refer to appropriate services, if needed. Items regarding nonadherence have options related to financial considerations. If the patient selects this as a need, it will trigger the pharmacist to explore a more cost-effective alternative (eg, cheaper medication and a blood glucose monitor).

### Digital Content Design and Usability

In addition, we are using software to produce animated videos to support content dissemination. For example, we will develop whiteboard animated videos and determine the types of animation preferred by patients during the acceptability and usability portion of the study (Phase II). Therefore, the qualitative portion of the study (Aim 1) will assist us in having an intervention that is usable and acceptable by patients and pharmacists.

### Integration Into Clinical Workflow

We are also hoping the health outcome results of the pilot study are favorable or provide further insight into developing a better understanding of how to improve the content and interface of the digital health apps. If successful, our goal with this project is that the outputs created are provided in an easy-to-read format for the provider, in which they can edit after they meet with the patient and can simply link the document with the patient’s electronic medical record. This would allow for a seamless transition and potentially reduce workload.

### Implications for Policy and Practice

If successful, this project may assist in supporting the Centers for Medicare and Medicaid Services Diabetes Strategy 2024 [61]. For example, it may assist with telehealth flexibilities for diabetes self-management training, increase support for social determinants of health in identifying adherence barriers (eg, transportation and cost), and potentially decrease readmissions or urgent care visits.

### Limitations

While several strengths have been discussed, the study is not without limitations. Limitations include a small sample size, and the current plan is to conduct the study at a single location. Therefore, the generalizability to other populations may remain unclear. In addition, there could be difficulties with technology among older adults (eg, aged ≥65 years); however, we will attempt to mitigate this risk by recruiting participants with T2D across the eligible older adult age range. In addition, there is no control group for the pilot study. Therefore, the significance of the results may be limited when assessing if the health outcomes are attributed specifically to the intervention or other confounding factors.

### Conclusions

In summary, this study explores a critical issue in developing technology that engages patients and addresses their specific needs. It also seeks to improve the way pharmacists can review patient information to identify barriers to medication adherence and promote patient well-being. The research aims to expand existing knowledge on how to improve CMM and optimize precision counseling. The findings of this study are expected to provide valuable insights to improve the delivery of care and health outcomes in older patients with T2D.

## Abbreviations

AEOUS: Adjectival Ease of Use Scale
CMM: comprehensive medication management
GNEOI: Gagne’s Nine Events of Instruction
HbA1c: Hemoglobin A1c
IMB: information-motivation-behavioral
IRB: Institutional Review Board
NIH: National Institutes of Health
PHQ-2: Patient Health Questionnaire–2
SUS: System Usability Scale
T2D: type 2 diabetes
TAM: technology acceptance model
